# Intraspecific variability of cadmium tolerance and accumulation, and cadmium-induced cell wall modifications in the metal hyperaccumulator *Arabidopsis halleri*

**DOI:** 10.1093/jxb/erv144

**Published:** 2015-04-06

**Authors:** Claire-Lise Meyer, Michal Juraniec, Stéphanie Huguet, Elena Chaves-Rodriguez, Pietro Salis, Marie-Pierre Isaure, Erik Goormaghtigh, Nathalie Verbruggen

**Affiliations:** ^1^Laboratory of Plant Physiology and Molecular Genetics, Université Libre de Bruxelles, 1050 Brussels, Belgium; ^2^Laboratoire de Chimie Analytique Bio Inorganique et Environnement, Institut des Sciences Analytiques et de Physico-chimie pour l’Environnement et les Matériaux (IPREM, UMR 5254), Université de Pau et des Pays de l’Adour, 64053 Pau cedex 9, France; ^3^Laboratory for the Structure and Function of Biological Membranes, Center for Structural Biology and Bioinformatics, Université Libre de Bruxelles, 1050 Brussels, Belgium

**Keywords:** Accumulation, *Arabidopsis halleri*, cadmium, cell wall, FT-IR, tolerance.

## Abstract

Certain molecular mechanisms of Cd tolerance and accumulation have been identified in the model species *Arabidopsis halleri*, while intraspecific variability of these traits and the mechanisms of shoot detoxification were little addressed. The Cd tolerance and accumulation of metallicolous and non-metallicolous *A. halleri* populations from different genetic units were tested in controlled conditions. In addition, changes in shoot cell wall composition were investigated using Fourier transform infrared spectroscopy. Indeed, recent works on *A. halleri* suggest Cd sequestration both inside cells and in the cell wall/apoplast. All *A. halleri* populations tested were hypertolerant to Cd, and the metallicolous populations were on average the most tolerant. Accumulation was highly variable between and within populations, and populations that were non-accumulators of Cd were identified. The effect of Cd on the cell wall composition was quite similar in the sensitive species *A. lyrata* and in *A. halleri* individuals; the pectin/polysaccharide content of cell walls seems to increase after Cd treatment. Nevertheless, the changes induced by Cd were more pronounced in the less tolerant individuals, leading to a correlation between the level of tolerance and the extent of modifications. This work demonstrated that Cd tolerance and accumulation are highly variable traits in *A. halleri*, suggesting adaptation at the local scale and involvement of various molecular mechanisms. While in non-metallicolous populations drastic modifications of the cell wall occur due to higher Cd toxicity and/or Cd immobilization in this compartment, the increased tolerance of metallicolous populations probably involves other mechanisms such as vacuolar sequestration.

## Highlight

A huge variability in Cd tolerance and accumulation exist within *A. halleri*, and the relationship between tolerance, accumulation, and edaphic type is not straightforward. Cd-induced cell wall modifications suggest various shoot detoxification mechanisms.

## Introduction

For more than a decade, a great deal of attention has been paid to metal hyperaccumulator plants, particularly to two *Brassicaceae*, *Arabidopsis halleri* and *Noccaea caerulescens*. In contrast to the majority of metallophytes that stored metals in root cells, these species possess the ability to accumulate and tolerate high metal concentrations in their shoots [>1% zinc (Zn) and >0.01% cadmium (Cd) in dry leaf biomass] (Krämer, 2010). They constitute powerful models to study the mechanisms of metal homeostasis, adaptation to extreme environments, and evolution of complex naturally selected traits. By means of quantitative trait locus (QTL) mapping, transcriptomic studies, and functional analyses, important progress has been achieved in our understanding of the mechanisms underlying metal tolerance and accumulation in *N. caerulescens* and *A. halleri* (reviewed by Verbruggen *et al.*, 2009; Krämer, 2010). A model mainly based on studies in *A. halleri* emerges for Zn tolerance and accumulation in which constitutive high expression levels of genes involved in root uptake (several *ZIP* members), root to shoot translocation (*HMA4* and *NAS2*), and vacuolar sequestration (*MTP1*) play a central role.

In contrast, the mechanisms underlying Cd tolerance in hyperaccumulators are still poorly understood. The *HMA4* gene was shown to be a major determinant of Cd hypertolerance and accumulation in *A. halleri* (Courbot *et al.*, 2007; Hanikenne *et al.*, 2008; Willems *et al.*, 2010). A high transcript level was also reported in *N. caerulescens* and explained, as for *A. halleri*, by genomic copy number expansion and altered *cis*-regulation (Ó Lochlainn *et al.*, 2011; Craciun *et al.*, 2012). Up to now, there have been no clear candidates to account for Cd uptake from soil and Cd unloading. In leaves of *N. caerulescens*, vacuolar sequestration seems to be the main mechanism for Cd detoxification (Küpper *et al.*, 2004; Ma *et al.*, 2005; Ebbs *et al.*, 2009). The extraordinary vacuolar sequestration capacity of some populations was explained by high transcript levels of the tonoplast Cd transporter NcHMA3 (Ueno *et al.*, 2011). There is no vacuolar transporter of Cd described in *A. halleri*. *MTP1*, which seems to be a strong Zn transporter (Kawachi *et al.*, 2012), as well as *HMA3* do not co-localize with the QTLs identified for Cd tolerance or accumulation (Courbot *et al.*, 2007; Willems *et al.*, 2010). X-ray absorption spectroscopy revealed that in *A. halleri* shoot Cd binds to carboxyl and/or hydroxyl groups provided by organic acids and/or cell wall (CW) components (Huguet *et al.*, 2012; Isaure *et al.*, 2015). The role of CW/apoplast in Cd trapping was recently supported by microfocused X-ray fluorescence (μXRF) on *A. halleri* leaves. In the mesophyll, which is one of the main storage tissues, Cd seems to be present in intra- and extracellular compartments at similar levels (Isaure *et al.*, 2015). It is well known that plant CWs are rich in compounds able to bind metallic cations. Preferential accumulation of Cd in this compartment was reported for aquatic species such as *Phragmites australis* or *Halimione portulacoides* (Jiang and Wang, 2008; Sousa *et al.*, 2008) as well as for terrestrial plants, in roots (collenchyma of *Salix viminalis*; Vollenweider *et al.*, 2006) and in leaves (mesophyll of *N. praecox* after CdSO_4_ treatment; Koren *et al.*, 2013).

The challenge is now to work towards a more comprehensive understanding of the genetic basis of Cd tolerance and accumulation. This means identifying and validating other candidate genes involved in metal uptake and detoxification in shoots and verifying whether the mechanisms previously identified are specific to the ecotypes analysed or widespread in the species. To reach this goal, a good understanding of the polymorphism of tolerance and accumulation abilities is crucial. While Zn tolerance and accumulation seem constitutive, a huge variability of these traits was found between and within populations of *A. halleri* and *N. caerulescens*. On average, the non-metallicolous (NM) populations are less tolerant and more accumulator than the metallicolous (M) populations (Meerts and Van Isacker, 1997; Bert *et al.*, 2000; Roosens *et al.*, 2003; Pauwels *et al.*, 2006). Analysis of differentiation of Zn tolerance with that of neutral molecular markers suggested that this trait has been increased in M *A. halleri* populations through selection on standing genetic variation within local NM ancestral populations (Meyer *et al.*, 2010). Variability of Cd tolerance and accumulation was investigated in seven *N. caerulescens* populations in controlled conditions. An inverse relationship between accumulation and tolerance has been reported in most populations, but exceptions were found for some populations (Roosens *et al.*, 2003). Up to now, knowledge about the distribution of Cd tolerance and accumulation in *A. halleri* populations is scarce. These traits are assumed to occur throughout the species, but only three M populations were studied in controlled conditions (Bert *et al.*, 2003; Cosio *et al.*, 2004; Zhao *et al.*, 2006; Przedpełska-Wąsowicz *et al.*, 2012).

*Arabidopsis halleri* is a clonal, self-incompatible and highly outcrossing perennial Brassicaceae with a disjunct distribution in central Europe and eastern Asia. In Europe, this species is found at low altitude in industrial sites polluted by Zn, Cd, and Pb (in Northern France, Poland, Germany, and Italy among others) and at moderate to high altitude on soils with low levels of metals. Phylogeographic studies have demonstrated independent colonizations of metalliferous sites in distant geographic areas and the existence of two allopatric divergent genetic units in Europe (NW and SE) and a hybrid zone (HZ, from Slovenia to southern Poland) (Pauwels *et al.*, 2012). Populations from the two genetic units seem to have experienced a long-term barrier to gene flow, whereas in the HZ a secondary contact probably occurred among previously isolated genetic pools (Pauwels *et al.*, 2012). In the last decade, *A. halleri* has become a model for studies on metal tolerance and accumulation. It is indeed the closest metal-tolerant relative of the model species *Arabidopsis thaliana* (Al-Shehbaz and O’Kane, 2002), sharing 94% coding sequence identity and high syntheny (Roosens *et al.*, 2008).

The aim of the present study is to investigate the genetic variability of Cd tolerance, accumulation, and shoot detoxification in M and NM *A. halleri* populations belonging to the different genetic units. In the natural range of Cd contamination, shoots seems to be the main storage site (Dahmani-Muller *et al.*, 2001; Zhao *et al.*, 2006). Quantitative variation was captured with a sequential growth test classically used to demonstrate the level of tolerance of populations (Schat and Ten Bookum, 1992; Pauwels *et al.*, 2006), and a single dose test with an exposure concentration that can be found in metal rich-habitats. Given that a part of Cd is bound to CWs in *A. halleri*, changes in CW composition triggered by Cd treatment in different populations were investigated using Fourier transform infrared (FT-IR) spectroscopy. This technique in conjunction with data compression methods [such as principal component analysis (PCA)] is a powerful tool to screen large numbers of plants in a species or from different species for CW-related phenotypes (Chen *et al.*, 1998; Carpita *et al.*, 2001; Mouille *et al.*, 2003; Wang *et al.*, 2012). This approach was successfully applied in the monitoring of the modifications to CW composition induced by biotic and abiotic stress factors (Martin *et al.*, 2005; Fernandes *et al.*, 2013).

## Materials and methods

### Plant material

Seeds were harvested in two regions where M and NM populations of *A. halleri* ssp. *halleri* could be found in close proximity: the North of Italy and the South of Poland–North of Slovakia (GPS co-ordinates are given in Table 1). The edaphic type (metallicolous or non-metallicolous) of the sampled populations was established according to the total concentration of Zn, Cd, and Pb in soil (see Bert *et al.*, 2002; Table 1). In addition, seeds were collected in the populations Auby (AU) and Langelsheim (LAN) which have been used as reference populations for genetic and molecular studies on metal tolerance and accumulation in *A. halleri* (Talke *et al.*, 2006; Courbot *et al.*, 2007; Hanikenne *et al.*, 2008; Sarret *et al.*, 2009; Frérot *et al.*, 2010, among others). These nine populations belong to the different genetic units of *A. halleri* ssp. *halleri* (NW, SE, and HZ according to Pauwels *et al.*, 2012). As control non-tolerant non-accumulating species, seeds from *A. thaliana* ecotype Col-0 and *A. lyrata* ssp. *petraea* originating from an uncontaminated site in the Czech Republic (Unhost, Central Bohemia; Macnair *et al.*, 1999) were used.

**Table 1.** *Geographic location and edaphic type of the investigated* Arabidopsis halleri *populations*

Name	Type	Localization	Habitat	GPS co-ordinates		Total concentration in soil (μg g^–1^)			
				N	E	Zn	Cd	Pb	pH
I28	NM	Val Paisco, Italy	Roadside, underwood	46°03’26.36	10°14’33.74	323^*a*^	5^*a*^	237^*a*^	5.8^*a*^
I30	NM	Sommaprada, Italy	Meadow	45°59’28.03	10°16’19.38	183^*a*^	4^*a*^	168^*a*^	5.8^*a*^
I16	M	Val del Riso, Italy	Meadow near a metallurgic plant	45°51’34.40	9°52’34.94	13 779^*a*^	68^*a*^	1517^*a*^	6.9^*a*^
SK2	NM	Kosica Bela, Slovakia	Meadow	48°46’10.20	21° 07’48.60	51^*b*^	<1^*b*^	26^*b*^	ND
PL22	M	Bukowno, Poland	Meadow near a metallurgic plant	50°16’58.08	19°28’43.38	3911^*c*^	27^*c*^	1047^*c*^	6.9^*c*^
PL15	M	Katowice, Poland	heap from Zn smelter	50°17’12.96	19°01’32.04	10 163^*c*^	68^*c*^	3109^*c*^	6.2^*c*^
AU	M	Auby, France	Meadow near a metallurgic plant	50°24’23.91	03°04’56.38	25 945	137	4364	7.7
LAN	M	Langelsheim, Germany	ND	51°56’34.08	10°20’56.40	1179^*d*^	14^*d*^	ND	ND

NM, non-metallicolous; M, metallicolous; ND, not determined.

^*a*^ In Decombeix (2011).

^*b*^ In Bert *et al.* (2002).

^*c*^ In Kostecka (2009).

^*d*^ In Deinlein *et al.* (2012).

### Sequential growth test of Cd tolerance

Seeds from each population were sown on sand in a controlled growth chamber (16h light d^–1^, 100 μmol photons m^–2^ s^–1^ irradiance, 20 °C day/18 °C night, and 70% humidity). After 4 weeks of growth, a maximum of 20 seedlings per population were transferred to 4 litre vessels filled with a modified Murashige and Skoog solution consisting of: K_2_SO_4_ (0.88mM), KH_2_PO_4_ (0.25mM), NaCl (10 μM), Ca(NO_3_)_2_ (2mM), MgSO_4_ (1mM), FeEDDHA (20 μM), H_3_BO_3_ (10 μM), ZnSO_4_ (10 μM), MnSO_4_ (0.6 μM), CuSO_4_ (0.1 μM), and (NH_4_)_6_Mo_7_O_24_ (0.01 μM). To ensure metal bioavailability, the pH of the solution was buffered using 0.25mM MES (2-morpholino-ethanesulphonic acid) adjusted to 5.8 with KOH. To minimize local environment effects, seedlings were randomly distributed in the vessels so that each population was represented by at least one individual in each vessel. Vessels were randomly distributed in the growth chamber and moved around once a week during change of nutrient solution. After 3 weeks in nutrient solution, the sequential test started following the method described in Bert *et al.*, (2003). Plants were sequentially transferred to increasing concentration of Cd: 10, 50, 100, 150, 200, 250, 300, 350, 400, and 450 μM CdSO_4_. At the end of each week, the roots of each plant were gently dried with tissue paper and the whole plant was weighed. Tolerance was determined as the lowest concentration at which no increase in fresh biomass was observed (effective concentration for 100% growth inhibition, EC_100_). Sample sizes varied from four to 16 and were determined by the availability of the seeds and the survivors during the acclimation step. The range of concentrations used in this test are far in excess of the metal contamination found in natural soil solution but were demonstrated to be useful to discriminate the tolerance of species, populations, or progeny (Schat and Ten Bookum, 1992; Bert *et al.*, 2003; Pauwels *et al*., 2005).

### Cd accumulation and mineral profile

After 4 weeks growth on sand, seedlings of each population were transferred to 4 litre vessels containing the nutrient solution. Seedlings and vessels were randomized as for the sequential test and the solution was changed each week during 3 weeks. Then, half of the seedlings were transferred to vessels containing 5 μM CdSO_4_. The sample size of each population in each condition ranged from four to 16 and was determined by the availability of the seeds and the survivors during the acclimation step. After 3 weeks, the plants were harvested, and the shoots were separated from the roots, washed with deionized water, and dried at 37 °C until constant weight. Root samples were not analysed due to their low biomass and the possible metal precipitation on the root surface. The Cd exposure concentration used in this experiment is in the natural range of Cd contamination and was described as the median toxic concentration in plants (Kopittke *et al.*, 2010). Further, after 3 weeks of contamination, the root to shoot translocation is not saturated for both *A. lyrata* and *A. halleri* populations (data not shown), allowing identification of different behaviours of accumulation. Before harvesting, the relative chlorophyll content of three leaves per individual was measured using a CCM-200 chlorophyll meter (Opti-Sciences, Hudson, NH, USA) which determines the relative content using dual wavelength optical absorbance (653nm and 931nm). Finally, the shoot dry weight (DW) was measured and the samples ground. Aliquots of 75mg of shoot material (DW) were digested in a 1:1 volume of H_2_O_2_ and HNO_3_ for 3h at 80 °C and filtered at 0.45 μm. Total Cd, Zn, Fe, K, Mg, and Ca in digests were measured after dilution using an inductively coupled plasma mass spectrometer (ICP-MS; CRC 7500cs; Agilent Technologies). Quality control for plant samples was based on the use of certified standard samples (spinach leaves: SRM 1570a–NIST).

### Preparation of cell walls

Individuals from five populations of the Cd accumulation experiment were used for further analysis of the CWs: M populations AU, I16, and PL22; NM population I28; and the control species *A. lyrata petraea*. These populations display contrasting levels of Cd tolerance and accumulation. Extraction of CWs from the shoots was carried out according to Zornoza *et al.* (2002) with minor modifications. Briefly, 100mg of dried ground samples were successively washed with 80% ethanol (three times), chloroform/methanol (2/1, v/v) (once), and acetone (three times). Between each step, the supernatant was eliminated by centrifugation at 1200g for 5min. The final pellets of CWs were dried at 67 °C overnight.

### Fourier transform infrared/attenuated total reflectance spectroscopy of the cell wall

The response of the CW to Cd in *A. lyrata* and *A. halleri* populations from different edaphic types was characterized using FT-IR spectroscopy. All measurements were carried out on a Bruker Equinox 55 FT-IR spectrometer (Bruker, Karlsruhe, Germany) equipped with a liquid N_2_ refrigerated mercury cadmium Telluride detector. All spectra were recorded by attenuated total reflection (Goormaghtigh *et al.*, 1999). A diamond internal reflection element was used on a Golden Gate Micro-ATR from Specac (Orpington, UK). The angle of incidence was 45 °. Around 0.5mg of dried CW material was resuspended in 20 μl of dionized water, and 1 μl of this mixture was deposited on the diamond crystal. The sample was quickly evaporated in N_2_ flux. In order to tackle CW modification, the FT-IR measurements were recorded between 1800cm^–1^ and 800cm^–1^. Each spectrum was obtained by averaging 256 scans recorded at a resolution of 2cm^–1^. For each sample, corresponding to an individual plant CW extract, three technical replicates were analysed. Overall, at least 12 spectra were collected for each population and treatment.

### Statistical analysis

In order to analyse differences in Cd tolerance among populations, the survival curves obtained from the sequential growth test were fitted to a sigmoidal dose–response curve with a variable slope by non-linear regression using GraphPad Prism version 6 (GraphPad Software, La Jolla, CA, USA). The equation used for the non-linear regression was a three-parameter logistic equation established as follows: P_i_=100/{1+10^[(logXb–X)B_i_]}^S_i_ where logXb=LogT50_i_+(1/B_i_) {Log[(2^(1/S_i_))–1]}. The variable ‘P_i_ represents the survival proportion of population i, ‘X’ is the concentration of Cd in the solution, ‘T50_i_’ is the concentration for which 50% of individuals from population i had reached their EC_100_, ‘B_i_’ is the slope factor, and ‘S_i_’ is the symmetry parameter. The standard error (SE) and 95% confidence intervals (CIs) were estimated by GraphPad Prism. Differences among populations were tested using extra sum-of-squares F test (GraphPad Prism), and differences between edaphic type using Wilcoxon–Mann–Whitney exact test (StatXact v.8, Cytel Studio, MA, USA). T50_i_ and B_i_ from distinct populations were declared significantly different if their 95% CIs did not overlap.

Results of the experiment at a fixed concentration of Cd were analysed using non-parametric exact tests (StatXact v.8) which make no assumptions about distributions and are suitable for small and/or unbalanced samples. Differences among populations were investigated using Kruskal–Wallis exact test followed by non-parametric post-hoc test for multiple comparisons according to Siegel and Castellan (1988). Differences between treatments (NC and C conditions) were tested using Mann–Whitney test. Correlations between DW, relative chlorophyll content, and concentration of Cd, Zn, Ca, Mg, Fe, and K were examined using the Spearman coefficient (GraphPad Prism).

FT-IR data were analysed with Kinetics (a custom-made program running under Matlab 7.1, Mathworks Inc.) as follows. The water vapour contribution was subtracted with 1562cm^–1^ - 1555cm^–1^ as the reference peaks. The spectra were then baseline-corrected over the whole spectrum and normalized for equal area between 1800cm^–1^ and 900cm^–1^. The spectra were also smoothed at a final resolution of 4cm^–1^ by apodization of their Fourier transform by a Gaussian line. In order to evidence spectral variations induced by Cd, the mean spectra of untreated plants from population i were subtracted from the mean spectra of Cd-treated plants from population i. The ‘difference spectra’ were thus obtained for the population i, which represent the actual modifications caused by Cd in this population. In order to test statistically the modifications induced by Cd, Student’s *t*-tests were performed at every wave number. To account for multiple testing, adjusted *P*-values were computed using the Bonferroni procedure (Sokal and Rohlf, 1995). To identify differences among populations, two complementary statistical analyses were performed. First, hierarchical cluster analysis was performed on the ‘difference spectra’ of the five populations. Groups with most similar data were built using Ward’s algorithm. This method, which is based on the similarity among group members with respect to many variables (Ward, 1963; Johnson and Wichern, 2002), allows hierarchical clustering of *n* groups with minimum loss of information. Secondly, PCA was used to pinpoint spectral contributions that explain most of the variance present in the data set (individual IR spectrum). PCA is an unsupervised statistical method that enables a reduction of variables by building linear combinations of variables that vary together (Johnson and Wichern, 2002).

## Results

### Intraspecific variability of Cd tolerance

**Fig. 1.**
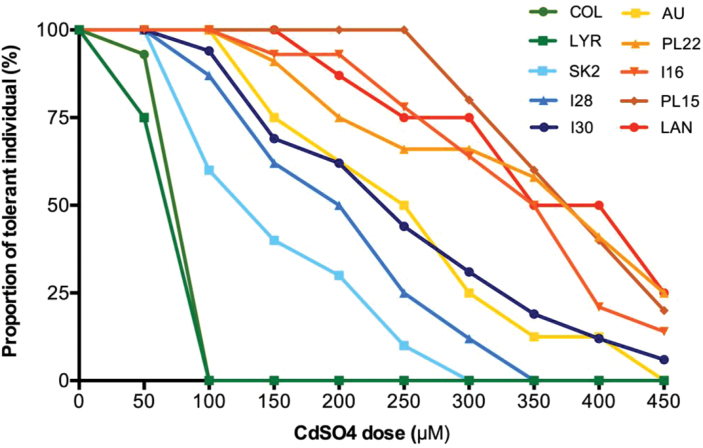
Cadmium tolerance of NM (SK2, I28, I31) and M (AU, PL22, I16, PL15, LAN) *A. halleri* populations and of *A. thaliana* (ecotype Col-0) and *A. lyrata petraea* (LYR). At each experimental dose, growth was encoded for each plant as a binary variable and interpreted as individual tolerance or sensitivity. *n*= 4–16. (This figure is available in colour at *JXB* online.)

In order to characterize intraspecific variability of Cd tolerance, a sequential growth test in hydropony was performed on *A. halleri* populations from M and NM sites located in the north and south of its distribution. As expected, individuals from the non-tolerant species *A. thaliana* and *A. lyrata* reached their EC_100_ at the first doses of exposure (i.e. 50 μM and 100 μM). Irrespective of the type of population, the range of Cd doses tolerated by *A. halleri* individuals was very large (Fig. 1). The first mortality events were observed at 100 μM for most populations, whereas some individuals remained alive at the highest dose (450 μM) in M populations and in one NM population. Besides this high variability, two interesting results have to be noted. First, the high proportion of individuals reaching their EC_100_ at 100 μM (like *A. thaliana*) in the NM population SK2 from Slovakia (four out of 10) and, secondly, the survival curve of the M population AU (France) which is similar to those of the NMs. All survival data were well fitted by dose–response curves (all *R* ^2^>0.97; see Table 2). Estimated values for T50_i_ (the concentration for which 50% of individuals from population i had stopped growing) were significantly different among populations (*P*<0.001) and the mean value for M populations was significantly higher than for NM populations (*P*=0.0357). Comparison of the T50 CIs confirmed that Cd tolerance increased from NM populations to M populationss (Table 2). The most sensitive population was SK2 (NM from Slovakia, T50 CI 111.8–143 μM), followed by I28 (NM from Italy, T50 CI 167.5–197.2 μM), I30 (NM from Italy, T50 CI 209.1–236.7 μM), and AU (M from France, T50 CI 217.3–254.8 μM). Other M populations (PL15 and PL22 from Poland, I16 from Italy, and LAN from Germany) showed overlapping T50 CIs (317.8–392.7 μM) which are significantly higher than those of the NMs (Table 2). The slope factor of the dose–response curves which estimates the range of within-population polymorphism did not reveal any difference between edaphic types.

**Table 2.** Estimations of T50 and slope factor obtained from fitting population survival curves to a sigmoidal dose–response model T50 values and slope factors are considered as estimations of the average tolerance of populations and of the within-population polymorphism, respectively.

Pop	Type	T50 (μM CdSO4)		Slope factor		*R* ^2^	*n*
		Estimated ±SE	95% CI	Estimated ±SE	95% CI		
I28	NM	188.7±1.03	175.5 to 202.9	–2.462±0.20	–2.937 to –1.986	0.99	8
I30	NM	230.1±1.02	215.4 to 245.7	–2.215±0.38	–3.131 to –1.300	0.99	16
SK2	NM	133.2±1.06	115.8 to 153.2	–2.004±0.61	–3.448 to –0.559	0.98	10
AU	M	235.3±1.03	217.3 to 254.8	–2,669±0.62	–4.145 to –1.193	0.98	8
PL22	M	352.5±1.03	324.6 to 382.7	–2.158±0.34	–2.981 to –1.335	0.97	12
PL15	M	379.2±1.01	368.5 to 390.1	–5.282±0.42	–6.289 to –4.276	0.99	5
LAN	M	370.9±1.02	347.6 to 395.8	–2.947±0.58	–4.319 to –1.574	0.97	5
I16	M	337.1±1.01	324.1 to 350.7	–3.830±0.29	–4.534 to –3.127	0.99	14

NM, non-metallicolous; M, metallicolous; SE, standar error; CI, confidence interval; n, sample size.

### Intraspecific variability of Cd accumulation

**Fig. 2.**
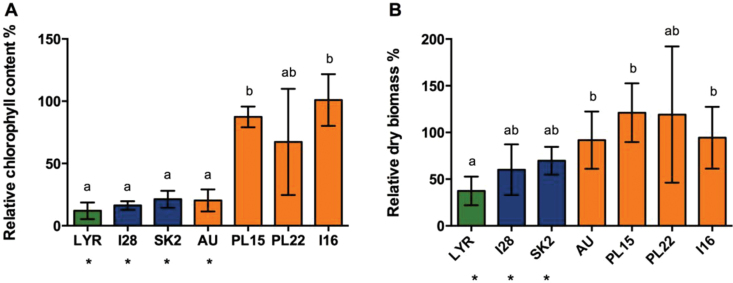
Effect of Cd on (A) chlorophyll content and (B) dry shoot biomass in hydroponically grown *A. halleri* NM (I28, SK2) and M (AU, PL15, PL22. I16) populations and *A. lyrata petraea* (LYR). Plants were cultivated for 3 weeks in a solution containing 5 μM CdSO_4_. Values are expressed relative to control conditions (0 μM CdSO_4_). Data are the mean ±SD, *n*=7, 10, 4, 10, 11, 7, and 16, respectively. Asterisks and letters indicate significant differences at the 5% level between treatments and among populations, respectively. (This figure is available in colour at *JXB* online.)

Six-week-old hydroponically grown plants were subjected to 0 μM (NC condition) or 5 μM CdSO_4_ (C condition) treatments during 3 weeks. Under NC conditions, all populations tested showed similar values for shoot dry biomass and leaf chlorophyll content (Supplementary Fig. S1 available at JXB online). After Cd treatment, signs of toxicity were observed in *A. lyrata petraea* (LYR), all NM *A. halleri* populations, and in the M population AU. These individuals displayed marked chlorosis, and the relative chlorophyll content decreased by 78–86% (Fig. 2; Supplementary Fig S1). Inhibition of shoot growth by Cd was only significant in LYR and the NM populations (Fig. 2). Hence, three different phenotypes were observed (see Supplementary Fig. S2): chlorosis and inhibition of shoot growth (LYR and NM populations I28 and SK2); chlorosis without an effect on shoot growth (M population AU); and unchanged chlorophyll content and shoot growth (M populations PL15, PL22, and I16).

**Fig. 3.**
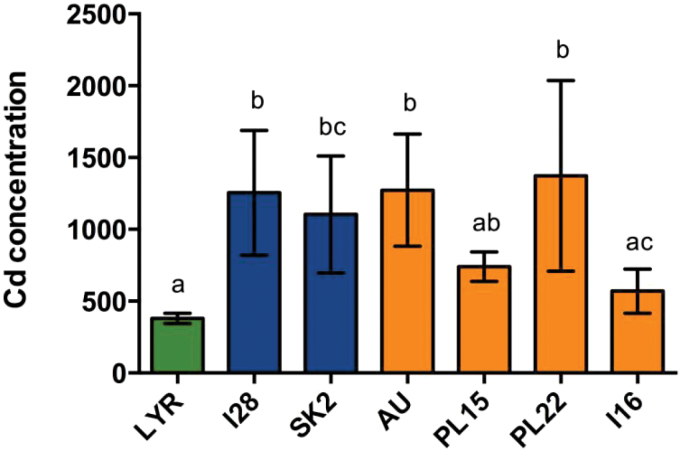
Cd concentration (μg ^–1^) in shoots of NM (I28, SK2) and M (AU, PL15, PL22, I16) *A. halleri* populations and *A. lyrata petraea* (LYR) after a 3 week exposure to 5 μM CdSO_4_. Data are the mean ±SD, *n*= 7, 10, 4, 10, 11, 7, and 16, respectively; letters indicate significant differences at the 5% level. (This figure is available in colour at *JXB* online.)

Large differences in shoot Cd concentrations were found among *A. halleri* populations (*P*<0.0001), with mean values ranging from 569 μg g^–1^ (I16) to 1372 μg g^–1^ (PL22). Apart from I16, the *A. halleri* populations displayed significantly higher shoot Cd concentration than LYR (376±59 μg g^–1^; Fig. 3). The ratio between shoot Cd concentration in AU and LYR (3.3) is in agreement with previous studies in soil (3.9; Willems *et al.*, 2010). The highest Cd concentrations and intrapopulation variability were found in the NM populations and in two M populations (AU and PL22), whereas in the populations accumulating less Cd the variability seems reduced (see SD on Fig. 3).

### Effect of Cd treatment on the ionome

**Fig. 4.**
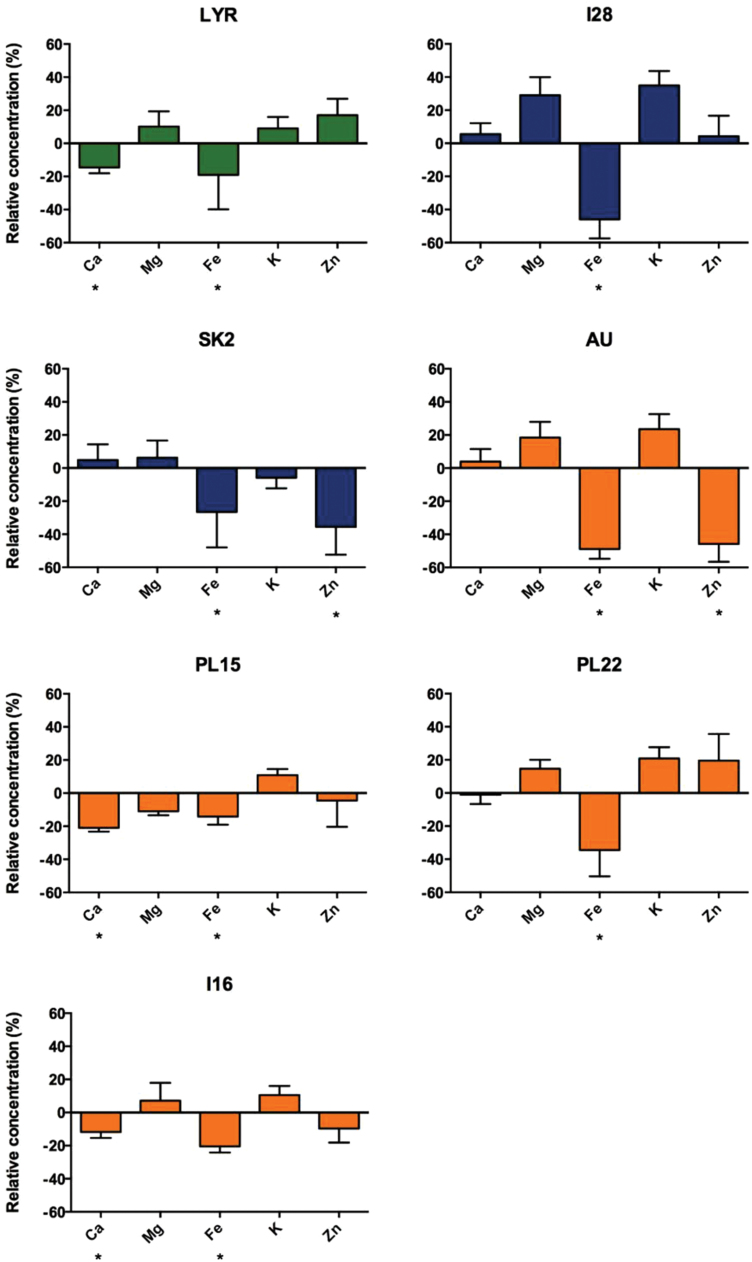
Changes in shoot mineral content of *A. halleri* NM (I28, SK2) and M (AU, PL15, PL22, I16) populations and *A. lyrata petraea* (LYR) after a 3 week exposure to 5 μM CdSO_4_. For each individual, the following ratio was calculated: (value in C condition - mean of the population in NC condition) / mean of the population in NC condition, and expressed in %. Data are the mean ±SD, *n*=7, 10, 4, 10, 11, 7, and 16; asterisks indicate statistical differences between treatments at the 5% level. (This figure is available in colour at *JXB* online.)

Under the NC condition, the mean Ca and Mg concentration in shoots did not differ among species or populations (Supplementary Fig. S3 at *JXB* online). As expected, the Zn concentration was significantly higher in all *A. halleri* populations (mean values ranging from 2930 μg g^–1^ to 6900 μg g^–1^) as compared with LYR (348 μg g^–1^). Significant differences among some populations were also found for Fe and K, but these differences were not specific to edaphic type or species (Supplementary Fig. S3). The 5 μM CdSO_4_ treatment leads to a decrease in Fe concentration in shoots of all populations (Fig. 4; Supplementary Fig. S4). This decrease was, on average, more severe in populations AU and I28 (–48% and –49% respectively; *P*<0.05). Shoot Ca and Zn concentrations were significantly affected by the Cd treatment in populations LYR and PL15 (–14% and –30% for Ca) and SK2, AU, and I16 (–43, –45, and –10% for Zn). The concentrations of Mg and K were similar in both treatments (Fig. 4). In *A. halleri*, the Cd concentration in shoots showed a significant correlation with Fe, Mg, and Ca (Supplementary Table S1; *r*= –0.443, *P*=0.0008; *r*=0.389, *P*=0.004; *r*=0.413, *P*=0.002; *n*=58). A strong negative correlation was found between the Cd and chlorophyll concentration (Supplementary Table S1; *r*= –0.718, *P<*0.0001). There was no significant correlation between Cd and Zn or between Cd and dry shoot biomass. Correlations between mineral nutrients were very similar in NC and C conditions (Supplementary Table S1).

### FT-IR analysis on cell wall

**Fig. 5.**
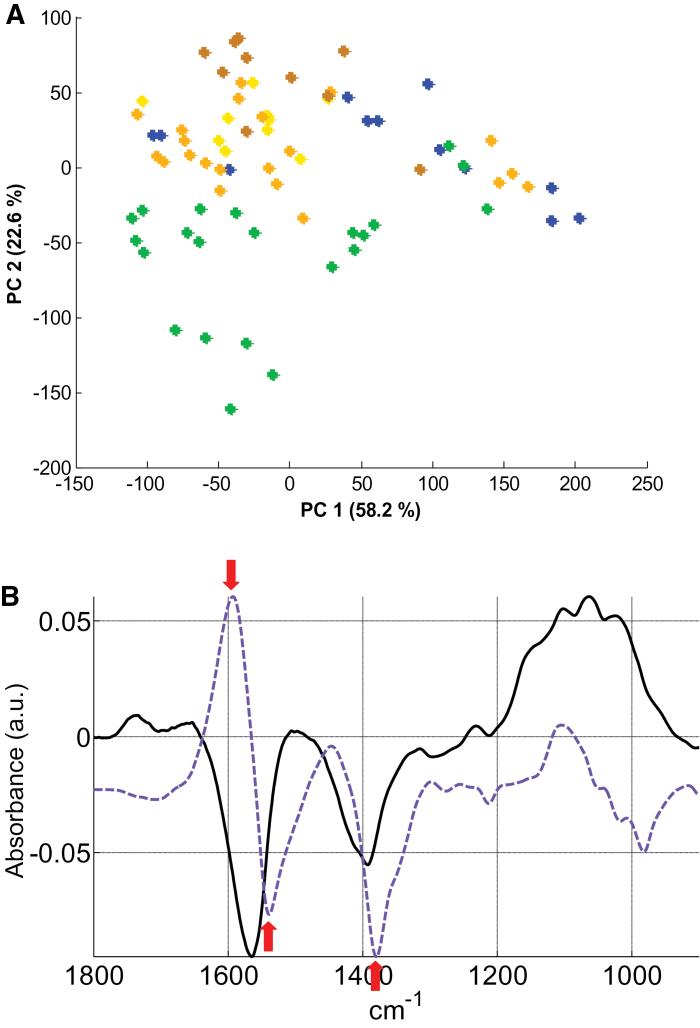
PCA analysis of FT-IR spectra obtained from all individuals in the NC condition (0 μM CdSO_4_). (A) Each dot is the projection of one spectrum on the first and second PCs. Green dots, *A. lyrata petraea*; blue dots, *A. halleri* NM population I28; from light to dark orange, *A. halleri* M populations AU, PL22, and I16. (B) Loading factor plots for PC1 (black line) and PC2 (blue line) explaining PCA clustering. Arrows point to the discussed wave numbers. Note that the curve of PC2 has been offset for better readability.

**Fig. 6.**
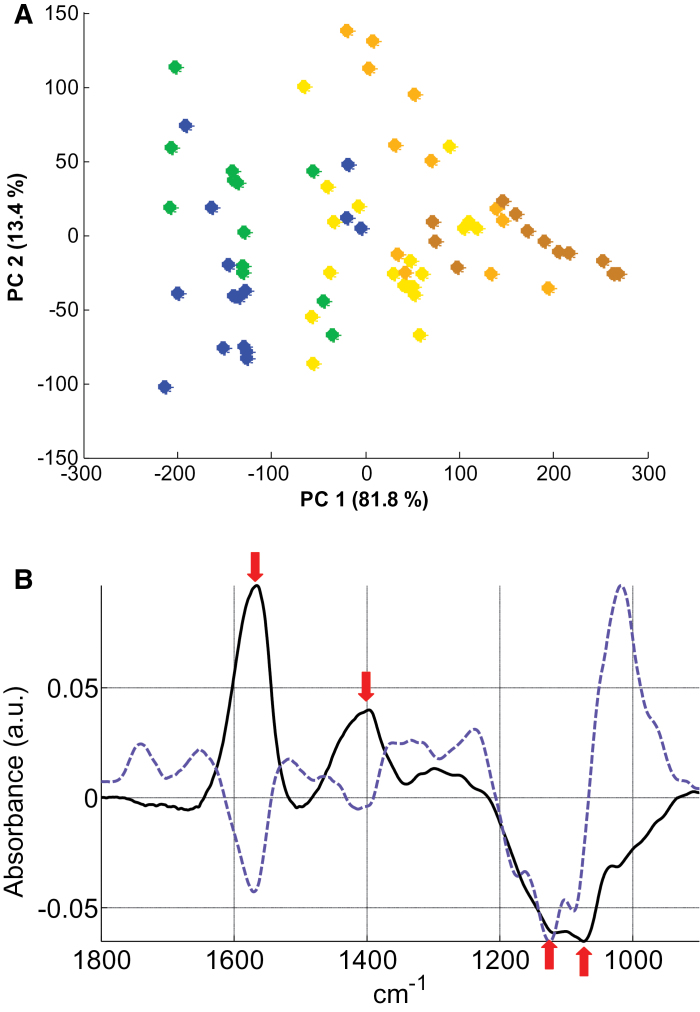
PCA analysis of FT-IR spectra obtained from all individuals in the C condition (5 μM CdSO_4_). (A) Each dot is the projection of one spectrum on the first and second PCs. Green dots, *A. lyrata petraea*; blue dots, *A. halleri* NM population I28; from light to dark orange, *A. halleri* M populations AU, PL22, and I16. (B) Loading factor plots for PC1 (black line) and PC2 (blue line) explaining PCA clustering. Arrows point to the discussed wave numbers. Note that the curve of PC2 has been offset for better readability.

The differences in CW composition among the populations LYR, I28, AU, PL22, and I16 were monitored in NC and C conditions (3 weeks at 5 μM CdSO_4_) by FT-IR spectroscopy applied to at least 12 samples per population and per treatment. For a clear analysis of the putative differences among populations, a multivariate analysis (PCA) was performed in each condition with the entire data set (Figs 5, 6). In the NC condition, principal component (PC) 1 which explained 58.2% of the total variance, was not useful to separate the samples. In contrast, PC2 (22.6% of the total variance) clearly differentiated the LYR samples from those of *A. halleri* (Fig. 5A). The PC2 loading factor plot (Fig. 5B) showed negative associations, with peaks corresponding to carboxylic acid groups (1540cm^–1^ and 1380cm^–1^ assigned to antisymmetric and symmetric -COO^–^ vibration, respectively; Séné *et al.*, 1994; Goormaghtigh *et al.*, 1994*a*; Jones *et al.*, 2005). These peaks are generally attributed to pectins with a low degree of methylesterification, which are rich in free carboxyl groups (Kakurakova, 2000). Proteins also give absorption at ~1550cm^–1^ (amide II N-H vibration; Goormaghtigh *et al.*, 1994*b*, 2006; Séné *et al.*, 1994). However, the peak of amide I expected at 1650cm^–1^ (C=O vibration; Goormaghtigh *et al.*, 1994*b*; Séné *et al.*, 1994) with an intensity ratio to amide II of ~2:1 was not detected. In the C condition, separation of the samples according to their Cd tolerance occurs on the basis of PC1 (81.8% of the total variance; Fig. 6A). The PC1 loading factor plot (Fig. 6B) showed positive associations, with peaks similar to those previously identified in NC conditions (1565cm^–1^ and 1396cm^–1^ assigned to -COO^–^ vibration; Séné *et al.*, 1994; Goormaghtigh *et al.*, 1994*a*; Jones *et al.*, 2005). Some negative peaks (1120cm^–1^ and 1075cm^–1^) were also observed in the region associated with ring vibrations as well as C-OH and C-O-C glycosidic bond vibration of polysaccharides (1200–900cm^–1^; Kakuracova, 2000). Since many complex vibrations of carbohydrates overlap in this region, unambiguous assignment of particular peaks is difficult (Kakuracova, 2000; Carpita *et al.*, 2001).

**Fig. 7.**
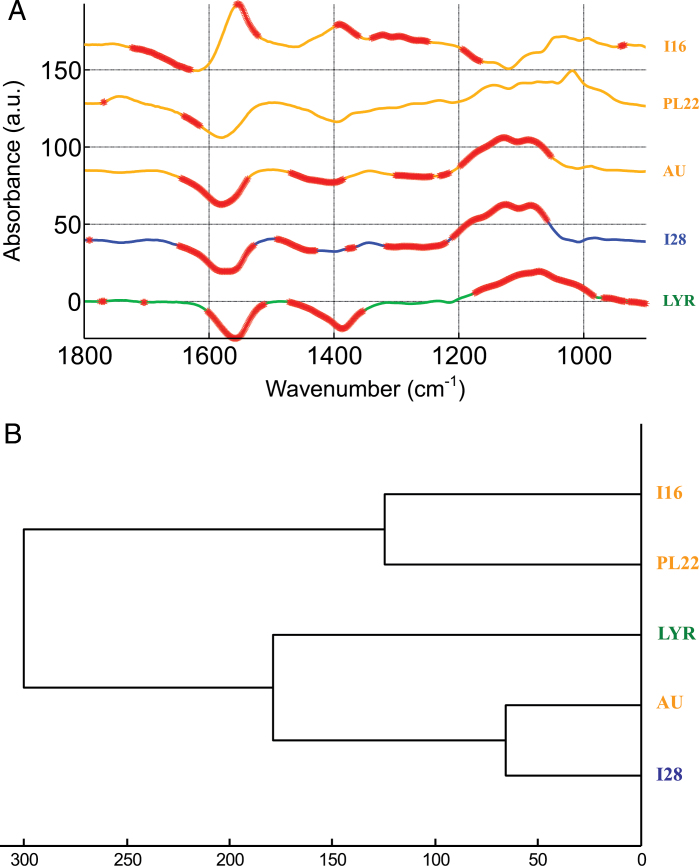
(A) Difference between the mean FT-IR spectra obtained at 0 and 5 μM CdSO_4_ for NM (I28) and M (AU, PL22, I16) *A. halleri* individuals and *A. lyrata petraea* (LYR). Student *t*-test was computed at every wave number with the significant level of 1%. Each marked wave number (thicker stars) indicates a statistically significant difference between treatments. Analysis was made on at least 12 FT-IR spectra per treatment and per population. Note that spectra have been offset for better readability. (B) Hierarchical classification of the difference spectra presented in (A). Classification was based on euclidian distances. (This figure is available in colour at *JXB* online.)

To acquire additional information on the response of populations to Cd treatment, a subtraction of the spectra was also performed (Fig. 7; Supplementary Figs S5–S9 at *JXB* online). Differences in spectra between NC and C conditions and Student *t*-test at each wave number were calculated for each population (Fig. 7A). It can be observed that spectral variations are very similar in LYR and *A. halleri* populations I28 (NM), AU (M), and PL22 (M). Nevertheless, most of the differences are not significant for the last population. A hierarchical classification of the difference spectra clearly shows the similarity between LYR, I28, and AU (Fig. 7B). The major differences between NC and C spectra (thicker lines in Fig. 7A) appear at wave numbers associated with carboxyl groups of unesterified pectin (negative peak at ~1550cm^–1^; Séné *et al.*, 1994; Goormaghtigh *et al.*, 1994*a*; Jones *et al.*, 2005) and with polysaccharides (several positive peaks between 1200cm^–1^ and 900cm^–1^). For LYR, strong differences were also observed in the region 1470–1355cm^–1^ (-COO^–^ vibration; Séné *et al.*, 1994; Goormaghtigh *et al.*, 1994*a*; Jones *et al.*, 2005). In order to identify the most significant peaks, PCA was performed on the total data set (NC+C) of each population (Supplementary Figs S5–S9). For LYR, I28, and AU, PC1 clearly differentiated the NC and C samples. As expected, the separation between NC and C was mainly explained by two negative peaks associated with carboxyl groups of unesterified pectin (1582–1560cm^–1^ and 1410–1390cm^–1^ depending on the population) and several positive peaks associated with polysaccharides. Separation between treatments was weaker for population PL22 and linked to negative peaks at 1567cm^–1^ and positive peaks at 1146, 1100, 1052, and 1020cm^–1^ (polysaccharides). The behaviour of the population I16 is different from that of the other tested populations. In the difference spectrum (Fig. 7), significant variations were found between 1725 and 1630cm^–1^, 1560 and 1520cm^–1^, and 1395 and 1165cm^–1^, but no significant changes were identified in the polysaccharide region (1200–900cm^–1^). Separation between treatments occurs on the basis of PC2 (33.7% of the total variance; Supplementary Fig. S9). PC2 loading factor plots showed major association with positive peaks at 1610cm^–1^ and 1550cm^–1^.

## Discussion

In order to investigate the natural variability of Cd tolerance and accumulation, and the mechanisms of shoot Cd detoxification of the model species *A. halleri*, sequential and single dose experiments were conducted with M and NM populations from the European genetic units NW, SE, and HZ (according to Pauwels *et al.*, 2012).

It was clearly demonstrated in this study that a huge polymorphism exists for Cd tolerance and accumulation between and within populations. Individuals displaying levels of tolerance similar to sensitive species were found in all tested NM populations, and the proportion of these individuals reaches 40% in the least tolerant population. As previously described for Zn (Pauwels *et al.*, 2006; Meyer *et al.*, 2010), on average the M populations are more tolerant than the NM populations whatever the genetic unit. The difference between edaphic types seems to be more pronounced than for Zn tolerance. Pauwels *et al.* (2006) have reported for Zn a continuum from the least tolerant to the most tolerant populations, while in the present study two groups of populations could be distinguished. However, this interpretation needs to be confirmed given the difference in sample size. Furthermore, a wide polymorphism was observed within NM populations, which suggests that the enhanced tolerance found in recently founded M populations may result from selection on standing genetic variation existing in NM populations, as previously proposed for Zn tolerance (Meyer *et al.*, 2010).

An unexpected and interesting result comes from the relatively low tolerance ability of the model M population AU. This population has been used in many experiments on Cd uptake and tolerance (Marquès *et al.*, 2002; Bert *et al.*, 2003; Cosio *et al.*, 2004), transcriptomics and proteomics (Craciun *et al.*, 2006; Farinati *et al.*, 2009), Cd localization (Huguet *et al.*, 2012; Isaure *et al.*, 2015), and quantitative genetics (Courbot *et al.*, 2007; Willems *et al.*, 2010). Both tolerance tests (sequential and at fixed concentration) showed a lower tolerance for this population as compared with the other M populations. After 3 weeks of culture with 5 μM CdSO_4_, all AU plants displayed chlorosis (Supplementary Fig S2 at *JXB* online) as pronounced as in the NM and *A. lyrata petraea* individuals. The bleaching of leaves could result from less efficient Cd detoxification mechanisms in the AU population compared with other M populations or from starvation of some essential elements. This population showed a strong competition between Cd and Zn as well as between Cd and Fe.

In the last few years, several studies have questioned the Cd hyperaccumulator status of *A. halleri* (Dahmani-Muller *et al.*, 2001; Bert *et al.*, 2002, 2003; Cosio *et al.*, 2004; Zhao *et al.*, 2006; Huguet *et al.*, 2012; Przedpełska-Wąsowicz *et al.*, 2012). Experiments conducted in controlled conditions on a few populations have shown that *A. halleri* is able to accumulate high amounts of Cd in shoots on soil and in hydroponic conditions, namely up to 5722 μg g^–1^ DW after 14 weeks treatment with 100 μM Cd (Küpper *et al.*, 2000). However, much lower Cd concentrations have been measured in *A. halleri* shoots under more realistic contamination levels. The question of intraspecific variability was only investigated *in situ* by Bert *et al.* (2002) on a few individuals (*n*=1–2) of 33 European populations. Although they concluded that hyperaccumulation is a property of the species, in metalliferous sites the proportion of individuals with Cd content above the threshold value used to define Cd hyperaccumulation (100 μg g^–1^ shoot DW; Brooks, 1998) was <20%. The present study is the first to investigate inter- and intrapopulation variability of Cd accumulation in controlled conditions. High levels of accumulation (>1100 μg g^–1^ DW) were reported in all NM populations tested and in one M population. Interestingly, the two other M populations coming from two different genetic units showed lower accumulation capacities which are close to the non-accumulating species *A. lyrata petraea*. Therefore, the results confirm that Cd hyperaccumulation is not constitutive at the level of the species and suggest that different strategies could have evolved in M populations to deal with high Cd content in soils.

Several experiments on *A. halleri* have reported Zn and Cd competition at the uptake or translocation sites (Küpper *et al.*, 2000; Zhao *et al.*, 2006; Ueno *et al.*, 2008; Przedpełska-Wąsowicz *et al.*, 2012). Conversely, Huguet *et al.*, (2012) did not observe a decrease in Zn accumulation after 3 and 9 weeks exposure to 20 μM CdCl_2_, suggesting that competition could depend on experimental conditions (Zn/Cd balance, exposure time, mineral composition of the solution, age of the plants) and/or genotypes. In the present experiment, the shoot Zn concentration was decreased by 5 μM CdSO_4_ treatment only in some populations (M and NM) and in very different proportions (9, 35, and 45%). This result suggests that despite the high transcript level of *AhHMA4* (Hanikenne *et al.*, 2013), some *A. halleri* populations could control the pathways of individual movements of these metals. In *N. caerulescens*, different pathways seem to exist, as highlighted by competition experiments (Küpper *et al.*, 2000; Roosens *et al.*, 2003). In addition, there was a possible competition between Cd and Fe in all populations tested as well as in *A. lyrata petraea*. This phenomenon was previously described by Küpper *et al.* (2000) and Zhao *et al.* (2006) in the population AU and one M population from Germany, but seems species-wide in the present experiments. In the non-tolerant and non-accumulator species, moderate to strong Cd-induced Fe deficiency is well known (Cohen *et al.*, 1998; Solti *et al.*, 2008). Indeed, given the similarity between Cd^2+^ and Fe^2+^ cations, the uptake of Cd from soil could occur via Fe^2+^ transporters such as IRT1 which is the major transporter responsible for iron uptake from soil and can transport Cd and Zn in addition to Fe (Vert *et al.*, 2002). However, expression of this gene in *A. halleri* is lower than in *A. thaliana*, thereby decreasing the disturbance of Fe homeostasis under metal excess (Shanmugam *et al.*, 2013).

The CW can play a major role in the immobilization of toxic metal ions by binding them to acidic pectins, histidyl groups, and negatively charged cellulose, or by constituting, through their carbohydrates, a barrier to metal uptake into the cytosol (Redjala *et al.*, 2009; Krzesłowska, 2011). Recently, the implication of the CW in shoot Cd detoxification was demonstrated in *A. halleri*. Using μXRF imaging, Isaure *et al.* (2015) showed in leaves of Auby individuals Cd location both inside the cells and at the rim, clearly supporting a CW/apoplast localization in addition to an intracellular pool. Hence, in order to better understand the polymorphism of Cd tolerance in *A. halleri*, differences in CW compositions in populations from different edaphic types were investigated using FT-IR. Although the analytical tools based on fractionation and fragmentation of CW glucans can give information of greater precision on constituent monosaccharides, they are not comprehensive in terms of complex architectural and compositional changes. FT-IR is very efficient to screen large numbers of plants and to analyse complex changes. Using this technique, it is not always easy to assign spectral peaks unambiguously to specific wall modifications, especially in lignified secondary CWs, but differences between species and modifications after treatment are clearly detectable. While the overall FT-IR results in the NC condition showed similar CW composition among *A. halleri* populations, a constitutive difference between *A. lyrata* and *A. halleri* was found, probably due to higher content of unesterified pectin in *A. lyrata* samples. These compounds, rich in free carboxyl groups, are well known to bind divalent and trivalent cations and were reported to limit the radial swelling stress induced by Cd (Douchiche *et al.*, 2010). Apart from the low accumulator I16, the effect of Cd on the CW composition was very similar between *A. lyrata petraea* and *A. halleri* individuals. CWs of plants growing in the presence of 5 μM CdSO_4_ seem to increase their polysaccharide content while reducing their unesterified pectin content probably due to compensation when a higher quantity of polysacchide occurs. The studies on the impact of Cd on plant CWs have reported an opposite response of pectin, namely an increase of the low-methylesterified fraction of pectin (Douchiche *et al.*, 2007; Fan *et al.*, 2011; Li *et al.*, 2015). Nevertheless, these studies investigated only the effect of Cd on CWs of roots and hypocotyl and not mature leaves. Polysaccharides and especially cellulose may contribute to metal tolerance and accumulation in various ways. Increased synthesis of cellulose could solidify and harden the CW, thus preventing metals from entering the cytosol (Degenhardt and Gimmler, 2000). A major role for cellulose in Cd binding was demonstrated by absorption kinetics of CW extracts from S*alix* roots (Chen *et al.*, 2013), and the increase of some polysacharides could contribute to metal tolerance by regulation of the glutathione-dependent phytochelatin synthesis pathway (Chen *et al.*, 2014). Interestingly, in the present study, the changes induced by Cd were more pronounced in the less tolerant individuals (including *A. lyrata petraea*), leading to a correlation between the level of tolerance and the extent of modifications (see Figs 6, 7B). This finding suggests a limited role for CWs in the hypertolerance of some *A. halleri* populations. In the more tolerant populations, other mechanisms of Cd detoxification (such as vacuolar sequestration) probably take place, whereas in the sensitive populations drastic modifications of the CW occur due to higher Cd toxicity and/or Cd immobilization in this compartment.

In conclusion, a complex picture emerges from the present results on Cd tolerance, accumulation, and Cd-induced CW modifications in *A. halleri*. All populations analysed are on average hypertolerant to Cd, but widely different degrees seem to exist within *A. halleri*, and the relationship between tolerance and accumulation is not straightforward. The fact that the M populations are on average more tolerant than the NM populations, together with the variability of tolerance among M populations, suggests evolution of this trait at a local scale. Furthermore, this hypothesis is supported by the different levels of Cd accumulation found in the M populations. Similar patterns were previously observed for the other model species *N. caerulescens* (Assuncao *et al.*, 2003; Roosens *et al.*, 2003). In the experiments reported here, the similar phenotypes found in M populations from different genetic units address the question of the convergent or parallel genetic evolution; that is, evolution of the same phenotype through similar or different genetic mechanisms (Arendt and Reznick, 2008; Stern and Orgogozo, 2009). Hence, these results highlight the importance of investigating Cd tolerance and accumulation mechanisms in populations other than the classically used Auby and Langelsheim.

## Supplementary data

Supplementary data are available at *JXB* online.

Figure S1. Dry shoot biomass and relative chlorophyll content in hydroponically growth *A. halleri* NM (blue bars) and M (orange bars) populations and *A. lyrata petraea* (green bars).

Figure S2. Different phenotypes of *A. halleri* populations and *A. lyrata* ssp. *petraea* after a 3 week exposure to 5 μM CdSO_4_.

Figure S3. Mineral concentration (μg g^–1^) in hydroponically grown *A. halleri* NM (blue bars) and M (orange bars) populations and *A. lyrata petraea* (green bars) after a 3 week culture in control solution (0 μM CdSO_4_).

Figure S4. Mineral concentration (μg g^–1^) in hydroponically grown *A. halleri* NM (blue bars) and M (orange bars) populations and *A. lyrata petraea* (green bars) after a 3week culture at 5 μM CdSO_4_.

Figure S5. Mean spectrum obtained at 0 (blue line) and 5 μM CdSO_4_ (black line) for *A. lyrata petraea* individuals and spectrum of the difference between these two average spectra (red line).

Figure S6. Mean spectrum obtained at 0 (blue line) and 5 μM CdSO_4_ (black line) for *A. halleri* individuals from the NM population I28 and spectrum of the difference between these two average spectra (red line).

Figure S7. Mean spectrum obtained at 0 (blue line) and 5 μM CdSO_4_ (black line) for *A. halleri* individuals from the M population AU and spectrum of the difference between these two average spectra (red line).

Figure S8. Mean spectrum obtained at 0 (blue line) and 5 μM CdSO_4_ (black line) for *A. halleri* individuals from the M population PL22 and spectrum of the difference between these two average spectra (red line).

Figure S9. Mean spectrum obtained at 0 (blue line) and 5 μM CdSO_4_ (black line) for *A. halleri* individuals from the M population I16 and spectrum of the difference between these two average spectra (red line).

Table S1. Correlations between the mineral concentrations, the concentration of chlorophyll, and the shoot dry biomass in the studied *A. halleri* individuals.

Supplementary Data
