# Supplementary material for: Intraspecific variability of cadmium tolerance and accumulation, and cadmium-induced cell wall modifications in the metal hyperaccumulator Arabidopsis halleri
Source: J Exp Bot. 2015 Apr 6;66(11):3215–27. doi: 10.1093/jxb/erv144 (PMC4449548; doi:10.1093/jxb/erv144)
Supplement: Supplementary Data [file supp_66_11_3215__index.html]

Intraspecific variability of cadmium tolerance and accumulation, and cadmium-induced cell wall modifications in the metal hyperaccumulator Arabidopsis halleri — Intraspecific variability of cadmium tolerance and accumulation, and cadmium-induced cell wall modifications in the metal hyperaccumulator Arabidopsis halleri — Supplementary Data 

# Intraspecific variability of cadmium tolerance and accumulation, and cadmium-induced cell wall modifications in the metal hyperaccumulator *Arabidopsis halleri*

## Supplementary Data

Data files

**Files in this Data Supplement:**

- Supplementary Data - Supplementary Data
