# Supplementary material for: Intraspecific variability of cadmium tolerance and accumulation, and cadmium-induced cell wall modifications in the metal hyperaccumulator Arabidopsis halleri
Source: J Exp Bot. 2015 Apr 6;66(11):3215–27. doi: 10.1093/jxb/erv144 (PMC4449548; doi:10.1093/jxb/erv144)
Supplement: Supplementary Data [file supp_erv144_jexbot143420_file001.pdf]

Intra-specific variability of cadmium tolerance, accumulation and Cd-induced cell wall modifications in the metal hyperaccumulator Arabidopsis halleri

Claire-Lise Meyer, Michal Juraniec, Stéphanie Huguet, Elena Chavez-Rodrigues, Piétro Salis, Marie-Pierre Isaure, Erik Goormaghtigh, and Nathalie Verbruggen

Table S1 : Correlations between the mineral concentrations, the concentration of chlorophyll and the shoot dry biomass in the studied *A. halleri* individuals. Upper part, in NC condition (0 µM CdSO4), *n* = 52 ; lower part, in C condition (5 µM CdSO4), *n* = 65.

	Cd	Zn	Fe	Mg	Ca	K	RCC	DSB
Zn	0.023		0.142	**0.455** **	**0.420** **	-0.111	0.066	-0.202
Fe	**-0.443** ***	-0.118		0.262	0.232	**0.366** *	0.115	-0.051
Mg	**0.389** **	**0.285** *	**-0.342** *		**0.595** ***	-0.180	-0.019	**-0.475** **
Ca	**0.413** **	**0.433** ***	-0.293	**0.601** ***		**-0.352** *	0.190	**-0.389** *
K	0.209	-0.037	**0.312** *	-0.260	-0.170		**-0.365** *	0.039
RCC	**-0.718** ****	0.102	**0.408** ***	**-0.295** *	0.141	**-0.356** *		0.258
DSB	-0.270	-0.204	0.129	**-0.273** *	0.118	-0.118	0.250	

RCC, relative chlorophyll content; DSB, dry shoot biomass; ****, $P < 0.0001$; ***, $P < 0.001$; **, $P < 0.01$; *, $P < 0.05$.

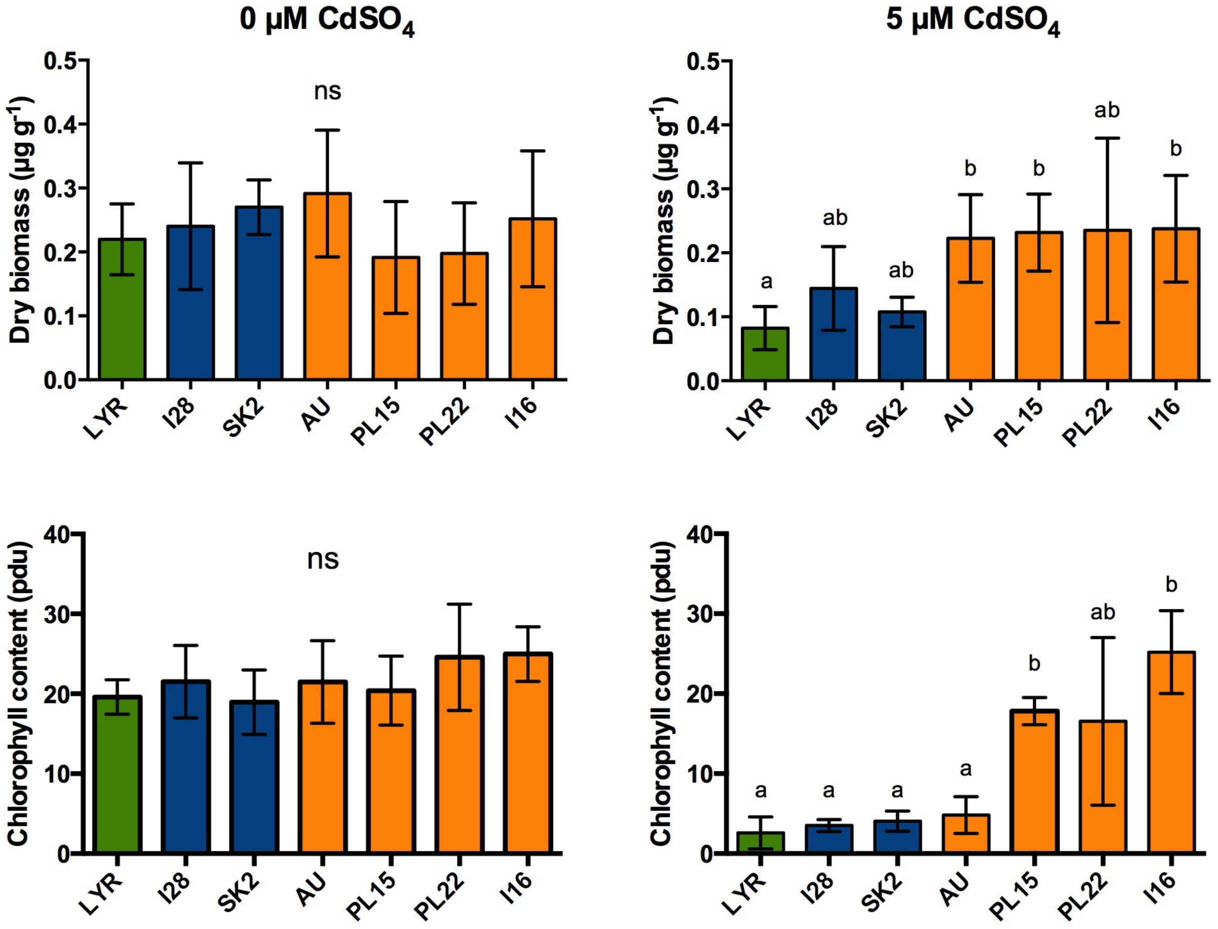

Figure S1: Dry shoot biomass and relative chlorophyll content in hydroponically growth *A. halleri* NM (blue bars) and M (orange bars) populations and *A. lyrata petraea* (green bars). Plants were cultivated for 3 weeks in control solution (0 µM CdSO4) or Cd contraminated solution (5 µM CdSO4). Data are mean ± SD, *n*= 3 to 16. Letters indicate significant differences at the 5 % level; ns, non significant.

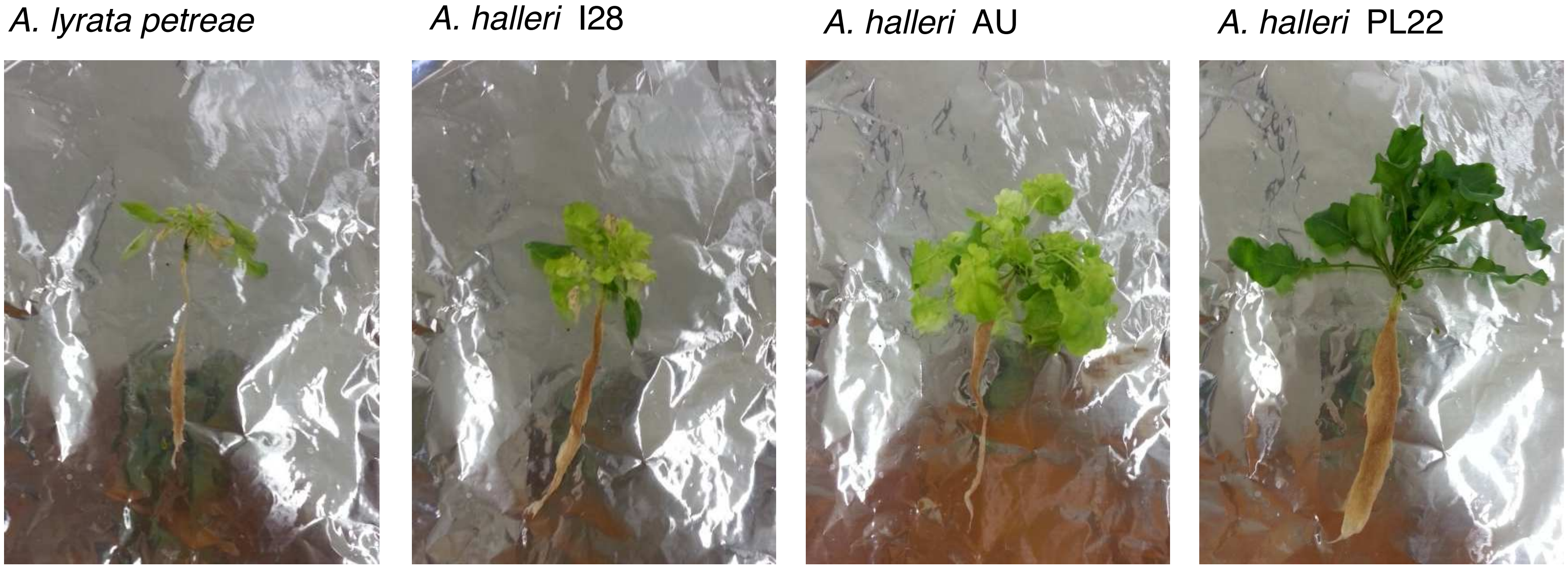

Figure S2: Different phenotypes of *A. halleri* populations and *A. lyrata* ssp *petreae* after 3 weeks exposure to 5 μM CdSO4. Scale is the same for all pictures.

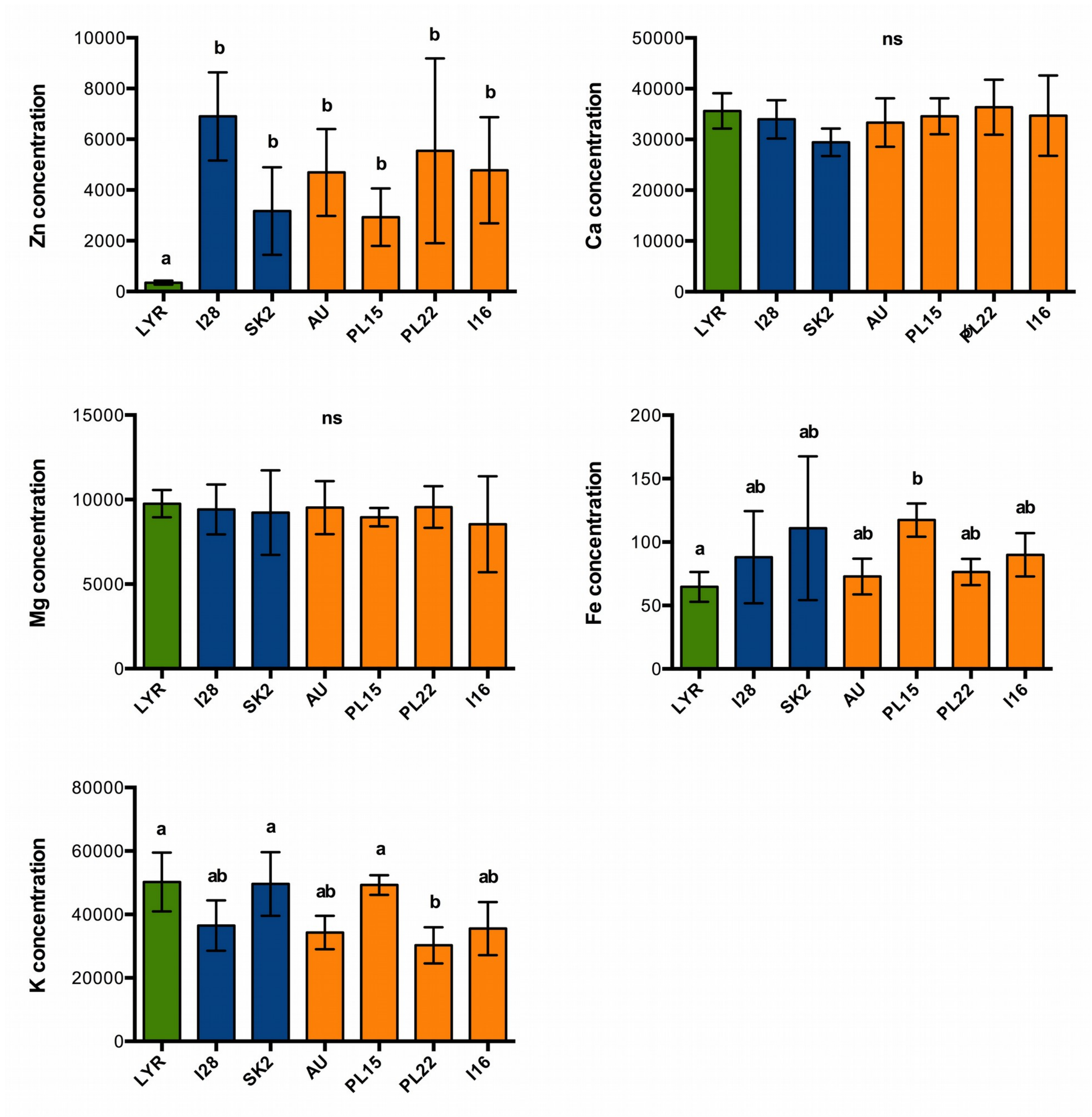

Figure S3: Mineral concentration (µg g-1) in hydroponically growth *A. halleri* NM (blue bars) and M (orange bars) populations and *A. lyrata petreae* (green bars) after 3-weeks culture in control solution (0 µM CdSO4). Data are means ± SD, *n* = 5 to 16. Letters indicate significant differences at the 5% level; ns, non significant.

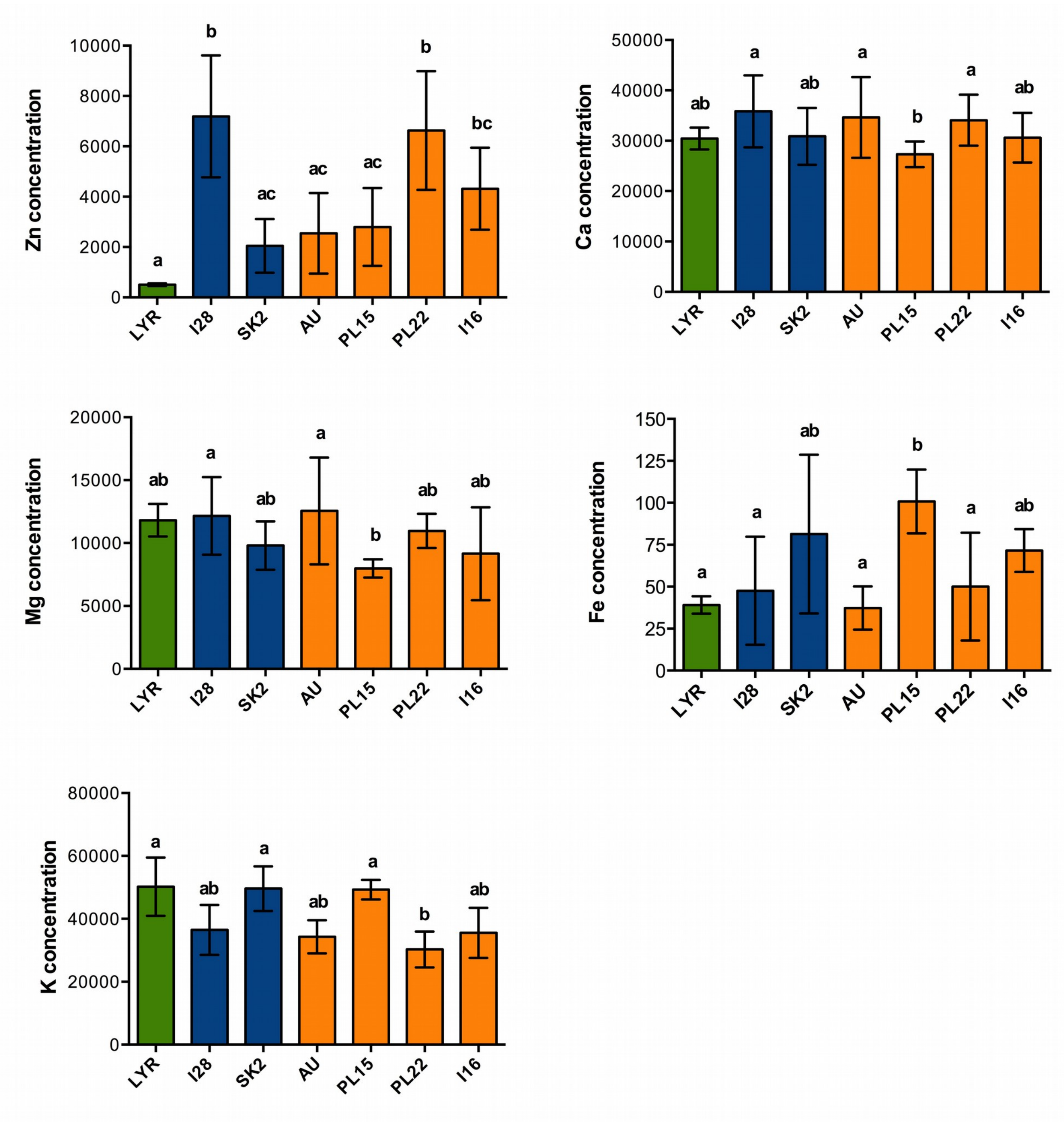

Figure S4: Mineral concentration (µg g-1) in hydroponically growth *A. halleri* NM (blue bars) and M (orange bars) populations and *A. lyrata petreae* (green bars) after 3-weeks culture at 5 µM CdSO4. Data are means ± SD, *n* = 4 to 16. Letters indicate significant differences at the 5% level.

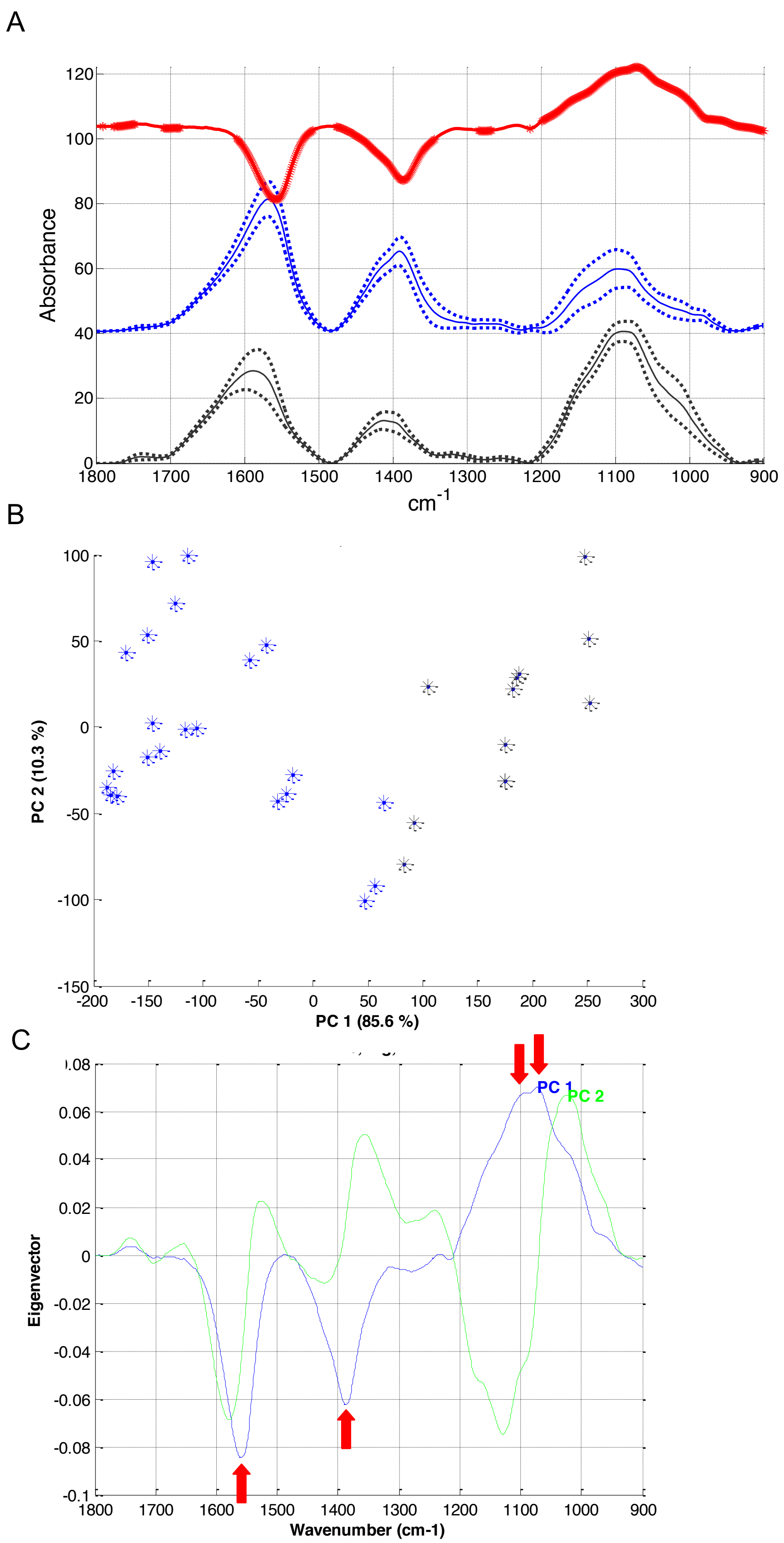

Figure S5: A, Mean spectrum obtained at 0 (blue line) and 5 µM CdSO4 (black line) for *A. lyrata petreae* individuals and spectrum of the difference between these two average spectra (red line). Thicker lines indicates wavenumbers statistically different according to a Student t-test (α=1%). Spectra have been normalized to the same arbitrary area between 1800 and 900 cm^{-1}. Note that spectra have been offset for better readability. B, Plot of the first and second PCs based on the FT-IR spectra obtained from *A. lyrata petreae* individuals grown in NC (blue dots) and C (red dots) conditions. C, Loading factor for PC1 (blue line) and PC2 (green line) explaining PCA clustering. Arrows point the discussed wavenumbers.

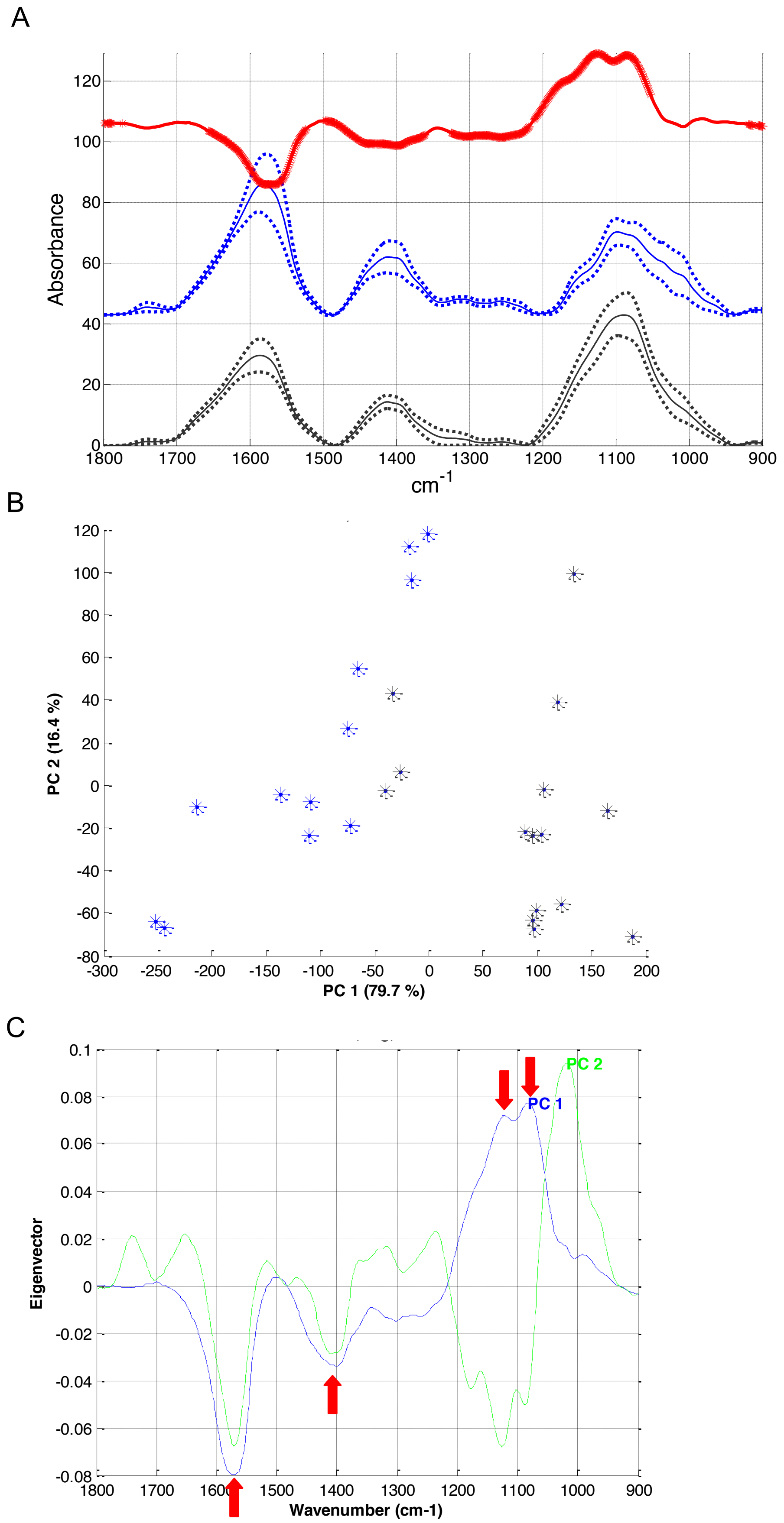

Figure S6: A, Mean spectrum obtained at 0 (blue line) and 5 µM CdSO4 (black line) for *A. halleri* individuals from the NM population I28 and spectrum of the difference between these two average spectra (red line). Thicker lines indicates wavenumbers statistically different according to a Student t-test (α=1%). Spectra have been normalized to the same arbitrary area between 1800 and 900 cm^{-1}. Note that spectra have been offset for better readability. B, Plot of the first and second PCs based on the FT-IR spectra obtained from *A. lyrata petreae* individuals grown in NC (blue dots) and C (red dots) conditions. C, Loading factor for PC1 (blue line) and PC2 (green line) explaining PCA clustering. Arrows point the discussed wavenumbers.

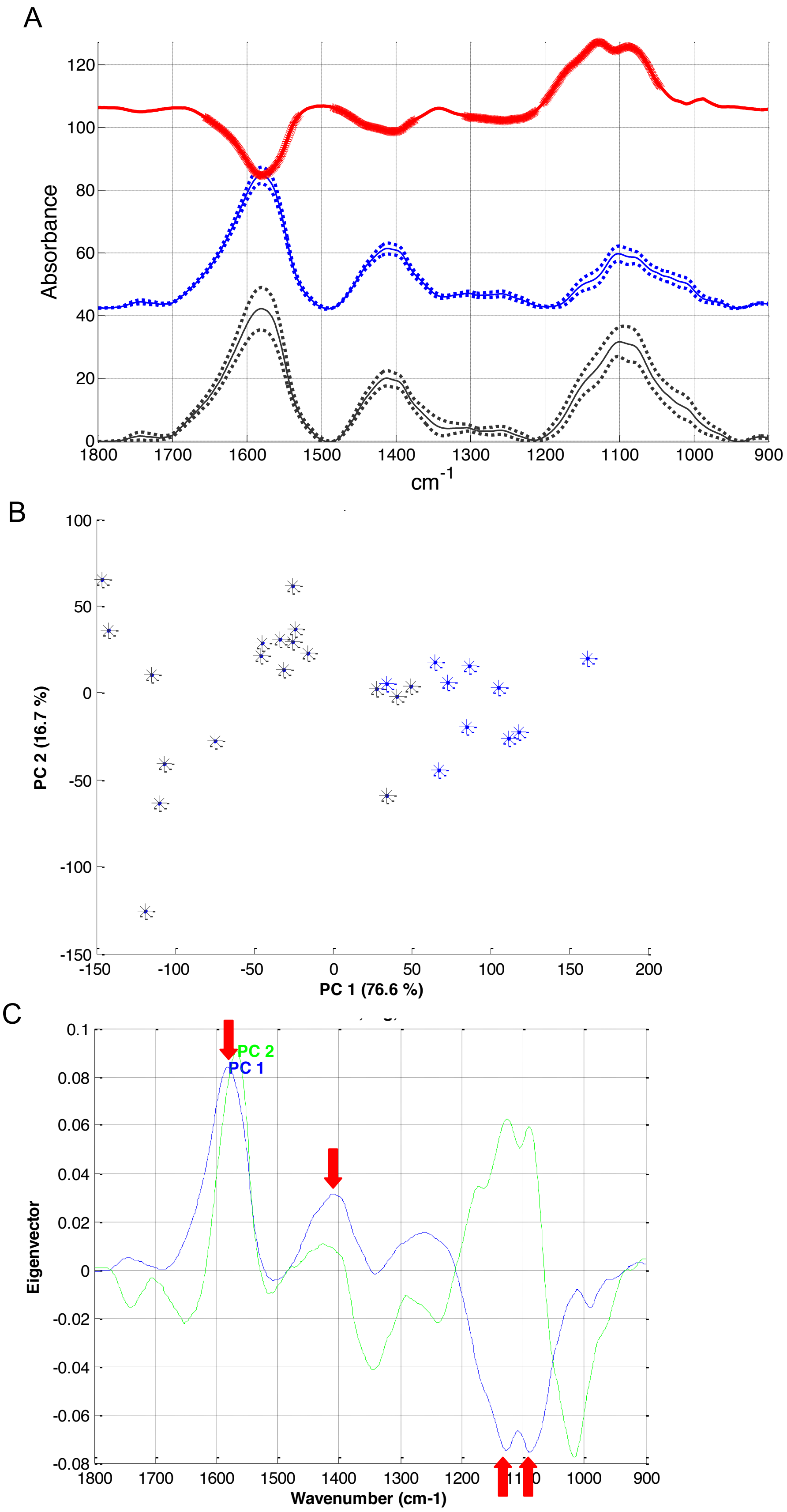

Figure S7: A, Mean spectrum obtained at 0 (blue line) and 5 μM CdSO4 (black line) for *A. halleri* individuals from the M population AU and spectrum of the difference between these two average spectra (red line). Thicker lines indicates wavenumbers statistically different according to a Student t-test (α=1%). Spectra have been normalized to the same arbitrary area between 1800 and 900 cm^{-1}. Note that spectra have been offset for better readability. B, Plot of the first and second PCs based on the FT-IR spectra obtained from *A. lyrata petreae* individuals grown in NC (blue dots) and C (red dots) conditions. C, Loading factor for PC1 (blue line) and PC2 (green line) explaining PCA clustering. Arrows point the discussed wavenumbers.

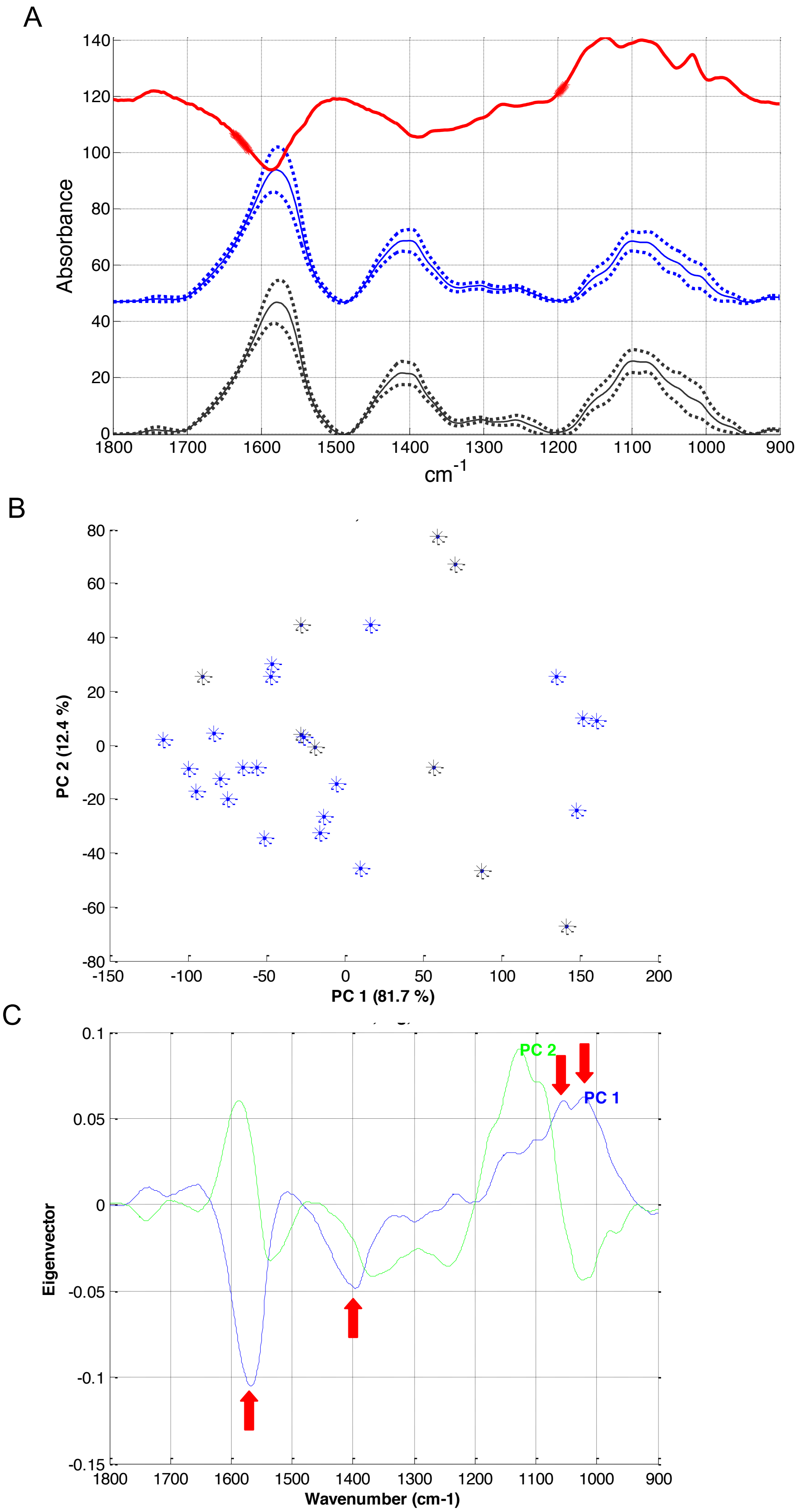

Figure S8: A, Mean spectrum obtained at 0 (blue line) and 5 µM CdSO4 (black line) for *A. halleri* individuals from the M population PL22 and spectrum of the difference between these two average spectra (red line). Thicker lines indicates wavenumbers statistically different according to a Student t-test (α=1%). Spectra have been normalized to the same arbitrary area between 1800 and 900 cm^{-1}. Note that spectra have been offset for better readability. B, Plot of the first and second PCs based on the FT-IR spectra obtained from *A. lyrata petreae* individuals grown in NC (blue dots) and C (red dots) conditions. C, Loading factor for PC1 (blue line) and PC2 (green line) explaining PCA clustering. Arrows point the discussed wavenumbers.

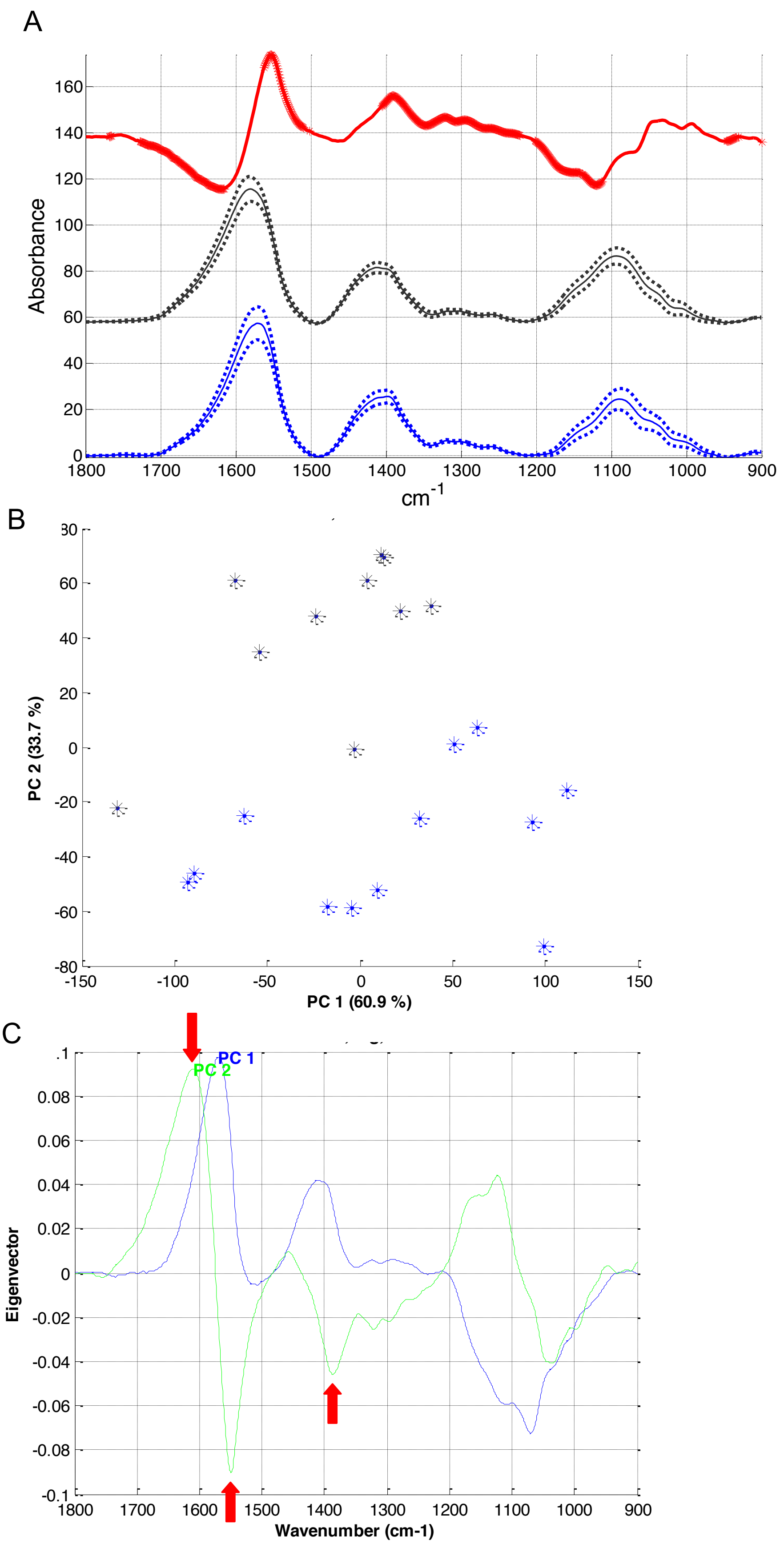

Figure S9: A, Mean spectrum obtained at 0 (blue line) and 5 µM $CdSO_4$ (black line) for *A. halleri* individuals from the M population I16 and spectrum of the difference between these two average spectra (red line). Thicker lines indicates wavenumbers statistically different according to a Student t-test (α=1%). Spectra have been normalized to the same arbitrary area between 1800 and 900 cm^{-1}. Note that spectra have been offset for better readability. B, Plot of the first and second PCs based on the FT-IR spectra obtained from *A. lyrata petreae* individuals grown in NC (blue dots) and C (red dots) conditions. C, Loading factor for PC1 (blue line) and PC2 (green line) explaining PCA clustering. Arrows point the discussed wavenumbers.
